# Information Pathways and Voids in Critical German Online Communities During the COVID-19 Vaccination Discourse: Cross-Platform and Mixed Methods Analysis

**DOI:** 10.2196/76309

**Published:** 2025-10-17

**Authors:** Silvan Wehrli, Anna-Maria Hartner, T Sonia Boender, Bert Arnrich, Christopher Irrgang

**Affiliations:** 1 Centre for Artificial Intelligence in Public Health Research, Robert Koch Institute, Nordufer 20, Berlin, 13353, Germany, 49 030187543732; 2 Medical Research Council Centre for Global Infectious Disease Analysis, Imperial College London, London, United Kingdom; 3 Department of Infectious Diseases, Public Health Service of Amsterdam, Amsterdam, The Netherlands; 4 Department of Health Sciences, Faculty of Science, Vrije Universiteit Amsterdam, Amsterdam, The Netherlands; 5 Department of Internal Medicine, Amsterdam UMC Location University of Amsterdam, Amsterdam, The Netherlands; 6 Amsterdam Institute for Immunology and Infectious Diseases (AI&I), Amsterdam, The Netherlands; 7 Amsterdam Public Health research institute (APH), Amsterdam, The Netherlands; 8 Digital Health - Connected Healthcare, Hasso Plattner Institute, University of Potsdam, Potsdam, Germany

**Keywords:** telegram, X, twitter, social media, querdenken, news, mainstream, health information, information ecosystem, COVID-19 vaccination

## Abstract

**Background:**

In Germany, the messaging app Telegram (Telegram FZ-LLC) served as a tool to organize protests against public health measures during the COVID-19 pandemic. A community of diverse groups formed around these protests, which used Telegram to discuss and share views outside of the general public discourse and mainstream information ecosystem. This increasingly included conspiracy theories and extremist content, propagated by sources that opposed the mainstream positions of the government and traditional media. While the use of such sources has been thoroughly investigated, the role of mainstream information in these communities remains largely unclear.

**Objective:**

We aimed to better understand the use of mainstream information, that is, from government actors and established media outlets, within critical Telegram communities in the context of the COVID-19 pandemic in Germany. We focused on the discourse about the COVID-19 vaccination, a key public health measure. As a central element of this study, we compared the Telegram discourse with the discourse on X (formerly Twitter, X Corp) and in online news—this cross-platform analysis aimed to put the results into a broader societal context.

**Methods:**

We analyzed Telegram, X, and news data between 2019 and 2023 for popular topics related to the COVID-19 vaccination discourse. We used a mixed methods approach, including text clustering for the exploration of popular topics, a 2-stage keyword filtering scheme for multitopic classification, link sharing analysis for assessing the prevalence of mainstream information, correlation-based time series analysis for measuring the similarity of discourse dynamics, and thematic analysis to examine the reasons for sharing information.

**Results:**

We identified 5 popular vaccination-related topics that were discussed on both Telegram and X, namely death, long COVID, measures in schools, mandatory vaccination, and virus variants. On average per topic, 58% (SD 5.2%) of Telegram posts and 21% (SD 4.9%) of X posts contained an external link. Of these posts containing external links, 11%‐35% of Telegram posts and 44%‐60% of X posts contained a mainstream link per topic. The correlations for week-to-week changes in discourse intensity between Telegram, X, and online mainstream news ranged from no positive association (coefficient <0.2) to strong positive relationships (coefficient >0.6) per topic. Finally, the thematic analysis resulted in 5 themes describing the usage patterns of mainstream information on Telegram and X. The identified themes are observing news, news comments, directed accusations, participation, and reference in discussion (only X).

**Conclusions:**

Mainstream information sources were part of the information mix within the analyzed critical Telegram communities. However, the role and prevalence of these sources varied. We argue that differences between platforms may indicate the existence of information voids, which pose a particular challenge in managing infodemics. These insights emphasize the importance of contextualized cross-platform analyses for understanding complex information pathways and their potential for targeted crisis communication.

## Introduction

The COVID-19 pandemic highlighted the critical role of information during public health crises. The spread of the virus, the uncertainty about the outcome of the pandemic, and the rapid changes in everyday life led to a high demand for information worldwide [1,2]. At the same time, this demand was met with a high supply of information of varying quality—a situation referred to as an infodemic [3]. Infodemics may pose significant health threats, such as when individuals become overwhelmed by the sheer volume of information and struggle to make informed health decisions [4]. In addition, the belief in false health information, including both unintentional misinformation and intentional disinformation [3,5], can undermine public health measures like the COVID-19 vaccination, which has saved countless lives in Europe [6,7]. Even though the necessity to understand and tackle infodemics has been widely acknowledged [8,9], it still remains a difficult challenge to quantify and understand infodemics [10]. As global pandemics could become more likely in the future [11,12] and with them public health infodemics, understanding infodemics remains relevant beyond current and past events. The information ecosystem is central to this, that is, the supply and demand of information by various actors and their interplay [13]. In such an ecosystem, different actors may be in (possibly reciprocal) relationships with each other. These relationships form information pathways, that is, the information flow between actors in the ecosystem [14].

During the COVID-19 pandemic, social media was an important information source alongside official announcements from government institutions and traditional media news outlets [2]. However, social media also became the focus of attention for spreading misinformation and disinformation as a medium with the ability to quickly spread information, possibly outside the established information ecosystem from authorities and others [9,15]. This proliferation may be facilitated by information voids, referring to situations where there is insufficient reliable information available on topics of public interest, creating opportunities for unreliable or misleading content to fill the gap [3,16]. Such gaps can arise, for example, when official sources are unable to meet the demand for information, especially in rapidly evolving crises, such as pandemics [3]. Apart from large social media platforms (eg, Facebook, Meta Platforms Inc or X, formerly Twitter, X Corp) [17], the messaging app Telegram (Telegram FZ-LLC) has shown particular potential for spreading false health information through a combination of circumstances [18-20]. Telegram’s channel function allowed individuals to address a large, public audience. In such channels, creators can post content that is accessible to a potentially unlimited number of subscribers, who can join freely [21]. In addition, Telegram did not remove users who had been removed from other platforms, such as Facebook for violating their platform rules [22]. In this context, Telegram is frequently referred to as a fringe platform [20,23-25], that is, a medium that allows almost unrestricted sharing of content, particularly extremist views and opinions that are marginalized by the rest of the public discourse [24]. Mainstream is used as a distinction from such fringe platforms, that is, for the dominant discourse outside of fringe groups [20,24] and social media platforms that restrict and remove extremist content [22,25]. We use this term similarly in this work, with mainstream representing opinions and views widely accepted by the general population. However, the concept of fringe platforms is not clearly defined [24] (other terms such as “alternative social media” are also used for similar concepts [22]). Furthermore, the use of Telegram does not necessarily imply a fringe discourse limited to extreme views outside the mainstream [26,27]. Ultimately, the use of Telegram may also be motivated by its variety of features that users find practical, such as the possibility to have private chats and consume news in 1 app [28].

In Germany, Telegram provided a platform for the organization of the Querdenken (literally “lateral thinking”) protests [20,29,30]. The protesters’ criticism was directed against the public health measures, which were generally supported by the broad public and leading media outlets [31,32], and not being allowed to express their critical positions during the COVID-19 pandemic [33]. The positions held by the protesters thus emerged as an alternative to the dominant public opinion in established, major media and from authorities [29]. The protests expanded across cities in Germany, with offshoots in Austria and Switzerland [20]. An active discourse formed around the protests on Telegram, consisting of a network of politically diverse groups, united by criticism of the pandemic response [33-36]. The discourse on Telegram increasingly attracted attention due to the proliferation of conspiracy theories about COVID-19 and politically extreme content (eg, from the far-right), supported by online sources outside of the mainstream information ecosystem [18,20,24,34,37]. These developments may suggest a general movement away from the mainstream discourse and information ecosystem. At the same time, however, the use of different information sources varied within different Telegram communities [35]. In addition, frequent sharing of articles from major German newspapers within Telegram channels [20,29,34,35] suggests a heterogeneous level of participation in the mainstream discourse. This asks for a differentiated and quantitative consideration of the heterogeneous communities’ interplay, which formed around the Querdenken protests. However, the role and impact of the mainstream information ecosystem on these communities and their relationship to broader societal conversations have remained unclear so far. Understanding this intricate relationship is crucial for the design and implementation of pandemic or infodemic risk communication, given the relevance of mainstream sources to the general public [2]. Ultimately, effective risk communication should reach the entire public.

Using a key example of the COVID-19 vaccination debate, we analyzed the information pathways between the Telegram communities around the Querdenken protests and the mainstream discourse. We focused on the role of information from governmental authorities and large media outlets, as these actors played a central role in the dissemination of information within the mainstream information ecosystem [2,32]. We considered 3 perspectives. First, we compared the prevalence of information from these mainstream sources on Telegram with the discourse on the social media platform X for popular vaccination-related topics, using techniques from natural language processing. Second, we applied time series analysis to the found discourse dynamics on the 2 platforms with regard to the publication of mainstream online news. Third, we examined the role of the shared mainstream information in the discourse on Telegram and X through qualitative data analysis. During these analyses, X served as a contrast to the Telegram discourse: X/Twitter actively tried to moderate misinformation and disinformation on the platform during most of the pandemic and the analyzed time period [22,25,38], respectively. In addition, the COVID-19 discourse on X in the German language was rather balanced, reflecting a broader spectrum of opinion in society [39-41].

The combination of multiple data sources and quantitative and qualitative methods revealed a complex relationship between the discourse on Telegram, on X, and in the mainstream information ecosystem, extending to communities that are particularly critical of the general public discourse. Our study thus emphasizes the potential of cross-platform analytics for understanding information pathways and information behavior during infodemics.

## Methods

### Data

#### Overview

We included data from public posts on Telegram, posts on X, and news headlines from the database of the Global Data on Events, Location and Tone (GDELT) project [42], all in the German language, between April 2019 and February 2023. The selection of the time period was based on the X data available to us, which covered the shortest period of time.

#### Telegram

For Telegram, we collected posts from public channels using the Telegram application programming interface, based on the work of Zehring and Domahidi [20], who analyzed a large number of Telegram channels linked to the Querdenken protests. Based on existing research, they first created an initial list of channels associated with the Querdenken protests or the far right. Then, they expanded this list to include channels whose content was frequently forwarded, that is, reposted, in the channels from the first selection. We note that, at the time of data collection (July 2024), 66 of the 558 channels were not accessible anymore (eg, due to deletion of the channel). We preprocessed the data similarly to Zehring and Domahidi [20] for their text analysis to obtain a dataset that was as similar as possible. Specifically, we removed non-German (as classified by fastText [43]) or empty posts and used the same final channel selection. The collected dataset contained original posts (without a reference to another Telegram post) and forwarded posts (or simply, forwards).

#### X (formerly Twitter)

The X data contained 1% of all public posts in the German language. This 1% sample could be streamed in real time directly from an application programming interface provided by X for the entire post volume on X (ie, for any language). Since this offer was accessible to a broad research community before it was terminated in February 2023 [44], it has served as the basis for numerous research projects [45]. We performed minimal text preprocessing and only refined the data quality through further language identification (using fastText [43]). The dataset contained original posts, replies, quotes, and reposts (formerly retweets).

We present the quantitative analyses below using different approaches to examine content-sharing mechanisms on Telegram and X. For Telegram, we included both original posts and forwards, as forwards represent a core mechanism for providing information within channels to their subscribers [46]. The Telegram dataset was specifically constructed by identifying channels through forward relationships, making forwards integral to understanding the information ecosystem in the studied dataset. For X, we excluded reposts from our analyses, similar to other studies [47,48]. X/Twitter content was susceptible to artificial amplification, for example, through reposts by automated accounts [49,50], as well as algorithmic content selection [51,52]. Excluding reposts allowed us to examine authentic content creation without the potential distortion introduced by artificial or algorithmic amplification. Conceptually, this corresponds to the scope of the study, as we focused on the information pathways, that is, the information flow, from mainstream actors to online platforms and communities, respectively.

#### News Headlines

The GDELT project [42] tracks news from a comprehensive set of online news sources worldwide to monitor and analyze the global news coverage. The project updates its databases continuously (every 15 minutes), using a dedicated server and crawler infrastructure in collaboration with a range of organizations, including Google News [53-55]. GDELT provides metadata on the crawled news and further analyses (such as sentiment analysis) through different datasets, which have been used by various studies in different scientific fields [54]. We used data from the Global Embedded Metadata Graph, which provides news article metadata on all news articles tracked by GDELT since 2018 [56]. Specifically, we extracted all news headlines in German from this dataset and their associated URLs and timestamps. We note that the extracted dataset contained duplicate URLs and duplicate news headlines (ie, from the same domain but published under different URLs), which we removed for the further analyses.

### Analytical Approach

#### Overview

We adopted an explanatory sequential mixed methods design [57]. Initially, we conducted quantitative data analysis, and the results from this phase informed the subsequent qualitative analysis. We focused on a selection of 5 popular topics related to the vaccination discourse on Telegram and X platforms, as described below.

#### Quantitative Analyses

##### Initial Content Filtering

In the first step, we filtered all datasets (Telegram, X, and news headlines) for vaccination-related content. We included all texts containing German words related to vaccination, names of COVID-19 vaccines, or their manufacturers (Table 1). We filtered the posts using a regular expression, which is provided in Listing S1 in Multimedia Appendix 1. We note that this filter considered compound words, different word forms, and hashtags. Based on our observation that both Telegram and X posts often contained inconsistent use of upper- and lowercase letters (eg, only lowercase letters), the filter did not distinguish between different capitalizations. In the course of the further study, we then only used these vaccination-related Telegram, X, and news headline datasets.

**Table 1.** Keywords used for filtering vaccination-related subtopics and the resulting sizes of data subsets.

Dataset	Keywords	Telegram posts (3,489,421)	X (formerly Twitter) posts (114,774,512)	Mainstream news headlines (6,134,824)
First-level filter (share in all data, %)				
Vaccination	Impfung, Vakzin (vaccine, vaccination), impfen, vakzinieren (to vaccinate); Bimervax, Comirnaty, Jcovden, Nuvaxovid, Spikevax, Sputnik V, Vaxzevria; AstraZeneca, BioNTech, CanSino, CureVac, Janssen, Johnson & Johnson, Moderna, Novavax, Pfizer, Sinopharm	426,142 (12.2)	2,135,912 (1.9)	129,812 (2.1)
Second-level filter (share in vaccination, %)				
Death	Tod (death), tot (dead), sterben (to die)	61,956 (14.5)	116,379 (5.4)	1642 (1.3)
Long COVID	Long-COVID, Post-COVID, Post-Vac, Langzeitfolge, Spätfolge (long-term consequence), Langzeitschaden (long-term damage)	4017 (0.9)	26,106 (1.2)	207 (0.2)
Mandatory vaccination	Impfpflicht (mandatory vaccination), Freiheit (freedom), Zwang (compulsion)	88,312 (20.7)	245,826 (11.5)	11,156 (8.6)
Measures in schools	Schule (school), Schüler (student), Lehrer, Lehrperson (teacher)	13,427 (3.2)	40,369 (1.9)	1949 (1.5)
Virus variants	Alpha, Delta, Omikron (Omicron)	9242 (2.2)	43,748 (2)	2308 (1.8)

##### Topic Exploration

As a second step, we used the text clustering framework BERTopic [58] to explore popular topics on Telegram and X. BERTopic uses numerical representations of text, derived from language models based on machine learning, as input to unsupervised clustering algorithms. These algorithms then form groups of semantically similar text documents into topics. We manually analyzed random samples to gain a deeper understanding of the topics’ content. We used BERTopic independently for both datasets to avoid skewing the clustering process toward popular X topics, given the different dataset sizes (Table 1). The implementation details regarding BERTopic, such as the choice of language model and other BERTopic model parameters, are provided in Multimedia Appendix 1. At this stage, we also expanded all short links in Telegram and X posts (as preprocessing for the text clustering and in preparation for further analyses), which is also described in Multimedia Appendix 1 (Section 2, with relevant sample code in Listings S2 and S3). Based on the topic exploration, we selected 5 topics from the 10 largest topics on both platforms for further analysis. Table S1 and Figure S1 in Multimedia Appendix 1 provide an overview of the 10 largest topics for both datasets.

##### Topic Filtering

We observed that posts, particularly on Telegram, frequently contained multiple vaccination-related topics within single posts. To address this, we developed a keyword-based classification scheme that could assign multiple topics to individual texts in a straightforward and interpretable manner. We selected keywords from BERTopic’s automatically generated topic descriptions, supplemented by manual review of random post samples per topic, prioritizing those that would capture the same topical content consistently across all datasets. This 2-stage keyword filter (following initial filtering for vaccination-related content) is comparable to the approach used by Purnat et al [59], who developed a keyword-based taxonomy to classify social media posts from various sources. We have presented the selected keywords in Table 1. We have provided the specific filter implementation, based on regular expressions, in Listing S4 in Multimedia Appendix 1. Again, the filter considered compound words, different word forms, hashtags, and different capitalizations. (Tables S2 and S3 in Multimedia Appendix 1 provide an overview of the overlap between different topics for Telegram and X posts). As a quality measure, we calculated the precision and recall of correctly classified Telegram posts, X posts, and news headlines based on random samples from each of the datasets. We have presented this analysis in Section 3, respectively, Table S4 in Multimedia Appendix 1. For the following analyses, we filtered the Telegram, X, and news headlines datasets using these topic-specific filters.

##### External Link Sharing

To investigate the prevalence of information from the mainstream discourse on Telegram and X, we measured the frequency of shared links from websites of governmental authorities and large media outlets in posts. To this end, we compiled a list of relevant website domains, with which we could then filter Telegram and X posts. For governmental authorities, we focused on the websites of state and federal governmental institutions. For news outlets, we prioritized nationally recognized privately and publicly owned media based on survey results and user metrics. This selection approximated the mainstream discourse by selecting sources with a particularly high reach in the general population. We provide a detailed explanation of our approach in Listing S1 in Multimedia Appendix 2, including sample code for extracting domains from links. Finally, we used the resulting list to limit the dataset of collected news headlines to the selected mainstream sources.

##### Time Series Analysis

To further explore the relationship between mainstream information and the discourses on Telegram and X, we analyzed the weekly frequency of posts and news headlines over time (ie, time series) for the selected subtopics. The aim of this analysis was to examine the relative importance of the subtopics on Telegram, X, and in mainstream news over time. The news headlines on specific subtopics served as a proxy for the extent of news reporting and mainstream information supply. This supplemented the previous analysis that did not provide any information about the temporal relationship between the platform-specific discourses. Specifically, we calculated the Pearson correlation coefficients between these time series (cross-correlation), which we selected for its intuitive interpretation and its invariance to scaling (as the analyzed datasets were of different sizes). We used the first-order differences (the changes of post and news volume from 1 week to the next week) to detrend the time series. Conceptually, this cross-correlation analysis allowed us to identify if an increase in the frequency of news headlines, that is, a particularly active discourse in mainstream sources, coincided with an increase in posts on Telegram or X for each of the selected subtopics.

### Thematic Analysis

In the final step, we qualitatively analyzed a sample of Telegram and X posts to better understand the reasons for sharing mainstream information. To this end, we used thematic analysis based on Braun and Clarke’s [60,61] framework. Thematic analysis is a qualitative research method used to organize and describe unstructured data through patterns (themes). These patterns reveal meaningful relationships and interpretations within the specific research context. In the context of this study, this meant the contextualization of mainstream information within the platform-specific discourse on Telegram and X. Particularly in connection with the Querdenken protests and the discourse on Telegram, previous research suggested a rather critical to negative view toward information from mainstream sources [29,34]. The aim of our analysis was to gain a more nuanced understanding of this view, also in contrast to the discourse on another platform (X). Thematic analysis thus provided a suitable complement to the quantitative analyses, which provided insights into the how and when of information behavior, but not into the why. Specifically, we randomly sampled equal numbers of unique posts with links to government, public media, and private media websites for each subtopic from the Telegram and X datasets. We analyzed a total of 1200 posts, which is within the range of similar studies in terms of quantity [41,62]. Since the X dataset is significantly larger, we sampled 3 times as many X posts. The initial data analysis was performed by the first author, who read the sampled posts repeatedly, noted interesting observations, and gradually abstracted similar observations into codes. The first author then structured these codes into initial themes, which were successively refined in consultation with the other authors.

### Ethical Considerations

All data used in this study were publicly accessible. Public posts on X and Telegram constitute public communication [63], given their specific orientation toward large audiences and unrestricted accessibility [21,64]. We did not analyze any information related to the authors of posts (eg, user IDs or user mentions). Importantly, all results refer to aggregated analyses, that is, were performed on the basis of groups of posts, and no conclusions were drawn about individual users, their behavior, or specific posts. Finally, while we do not publicly share any of the analyzed data, we outline the availability of the used data sources in the “Data Availability” section.

## Results

### Overview

For the following analyses, we selected 5 popular vaccination-related subtopics based on the initial exploration of topics on Telegram and X. To this end, we filtered all posts using vaccination-related keywords. Then, we used text clustering for topic exploration and developed a keyword filtering scheme for each selected subtopic, with which we filtered the vaccination-filtered datasets for a second time (cf Methods). The selected subtopics, their size, and the associated keywords used for the classification are summarized in Table 1. The keyword filtering considered compound words, different word forms (eg, different declinations), and hashtags. The filtering was case-insensitive. The implemented filter is specified in Multimedia Appendix 1. For Telegram, posts include original posts and forwards. For X, posts include original posts, replies, and quotes.

On Telegram, the subtopic of death was particularly present, with posts discussing deaths as a result of vaccination. By comparison, on X, deaths were also discussed as fatal consequences of COVID-19, with vaccination providing effective protection. Another prominent subtopic on both platforms was mandatory vaccination, in particular, posts discussing the likelihood of its implementation. This topic also included posts discussing the different rights of vaccinated and unvaccinated people as a result of measures relating to vaccination. Telegram posts expressed strong opposition to any form of mandatory vaccination, while X posts also expressed approval for such mandates. These opposing views on vaccination, as a health risk and a threat to personal freedom or as a protective measure and a necessity, were present in all of the analyzed subtopics. While posts on Telegram had an exclusively negative view on vaccinations, the analyzed X posts overall showed a more balanced view between perceived risks and benefits. This tendency was supported by the higher co-occurrence of death and mandatory vaccination with the other subtopics on Telegram compared to X, with rates up to 5 times higher (Tables S2 and S3 in Multimedia Appendix 1). The subtopic of long COVID discussed potential long-term health damages after a COVID-19 infection or COVID-19 vaccine. Telegram posts exclusively linked these effects to vaccination while X discussions also recognized vaccines as a protective measure. Similarly, for the subtopic of virus variants, which was addressed, they questioned the effectiveness of vaccines against emerging variants. Finally, the subtopic of measures in schools discussed the health and social effects of vaccinations, especially the necessity of measures and the relationship between vaccinated and unvaccinated individuals in schools.

### Prevalence of Mainstream Information

To assess the relationship of information between the mainstream discourse, that is, the prevalent discourse in the public debate, Telegram, and X, we analyzed the frequency of shared links from 63 domains of government institutions, 12 public media outlets (state ownership), and 47 private media outlets (private ownership) from Germany, Austria, and Switzerland. We selected these sources because of their wide reach among the general population. This included, among others, the websites of public health institutes (eg, Robert Koch Institute), of public broadcasters (eg, Tagesschau), and of newspapers and news magazines (eg, Der Spiegel). We provide a full list of all included sources in Table S1 in Multimedia Appendix 2.

**Figure 1.**
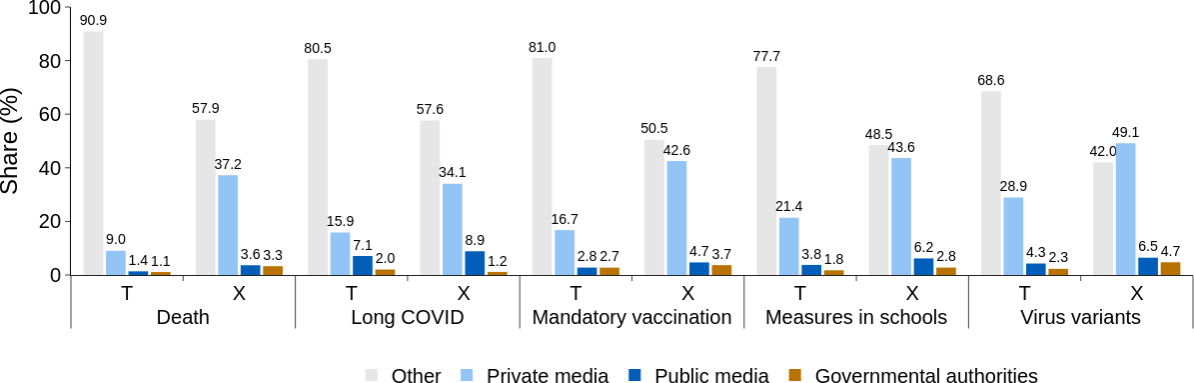
The percentage of posts that contain at least 1 link to information from governmental authorities, public or private media, or to some other source, relative to the number of all posts for Telegram (T) and X with at least one external link (a link that leads to a website outside the source platform).

On average, 58% (SD 5.2%) of Telegram posts and 21% (SD 4.9%) of X posts per topic contained at least 1 external link, which is a link that leads to a website outside of the platform from where it was shared. Considering only posts with external links, the prevalence of the examined sources varied per topic (Figure 1). The sum of percentage points in Figure 1 for a specific topic and platform may exceed 100% as Telegram and X posts may contain multiple links. For Telegram, the shares of the examined sources varied from roughly every tenth post (death) to every third post (virus variants). For X, the shares were consistently higher, and for some topics (eg, virus variants), at least half of the posts contained at least one of the examined links. The majority of these shared links pointed to private media outlets, followed by links to public media and government institutions, similarly for Telegram and X. An overview of the most shared domains is provided in Figure S1 in Multimedia Appendix 2 (along with an overview of the most shared top-level domains in Figure S2 in Multimedia Appendix 2). We note that, while posts may contain multiple links, only 0.5% (22/4056) of Telegram posts and 0.2% (20/8348) of X posts linked to 2 or more of the examined categories.

### Discourse Dynamics

We examined how the discourse intensity varied across Telegram, X, and mainstream news over time by calculating Pearson cross-correlations between the weekly number of Telegram and X posts, respectively, and news headlines. The news time series was restricted to headlines from the mainstream sources discussed in the section “Prevalence of Mainstream Information”. We provide detailed results in Tables S1 and S2 in Multimedia Appendix 3. We classified correlations as follows: no association (0.0-0.20), weak (0.20-0.40), moderate (0.40-0.60), and strong (>0.60).

Long COVID showed no association to weak relationships across all datasets, while death-related content correlated weakly. The topic measures in schools showed weak to moderate relationships, and virus variants showed moderate to strong correlations. Mandatory vaccination showed strong associations across all 3 datasets. Notably, correlations were consistently positive and stronger between Telegram and X than between either platform or mainstream news.

**Figure 2.**
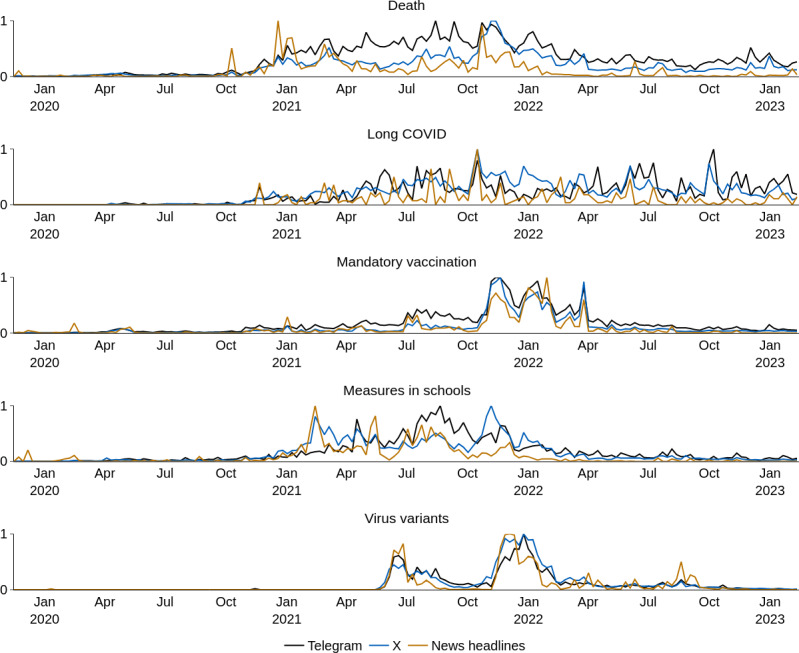
Weekly number of posts, respectively, mainstream news headlines.

Visual inspection of the time series provides insight into the observed correlation patterns (Figure 2). To enable comparison between time series of different magnitudes and focus on relative importance over time, weekly numbers are min-max normalized in Figure 2 (showing values relative to each series’ maximum). The visualization shows a section of the entire time series (April 2019-February 2023). Death-related discourse showed some synchronized peaks in early 2021 across all platforms, consistent with the weak correlations found, as the synchronized activity was concentrated in a relatively brief period. The long COVID discourse appeared scattered and varied across platforms throughout the observation period, with almost no synchronized peaks, explaining the weak correlations observed for this subtopic. The mandatory vaccination discourse remained low in intensity until late 2021, when sudden increases in discourse intensity occurred across all platforms, directly corresponding to the strong correlations found for this topic. Measures in schools displayed periodic synchronized activity primarily in 2021, aligning with the weak to moderate correlations observed. Virus variants showed distinct, sharp synchronized peaks concentrated in mid-2021 and early 2022, with all platforms showing simultaneous activity during these periods, which accounts for the moderate to strong correlations found for this subtopic.

In a second set of calculations, we analyzed intraplatform correlations for subtopics on Telegram and X, comparing all posts with those linked to mainstream sources, that is, measuring how mainstream-linked posts aligned with the broader platform discourse. Correlations were positive for all subtopics. Telegram showed a weak relationship for long COVID, moderate relationships for death and measures in schools, and strong relationships for mandatory vaccination and virus variants. Except for long COVID, X showed strong correlations for all subtopics.

### Purpose of Sharing Mainstream Information

**Figure 3.**
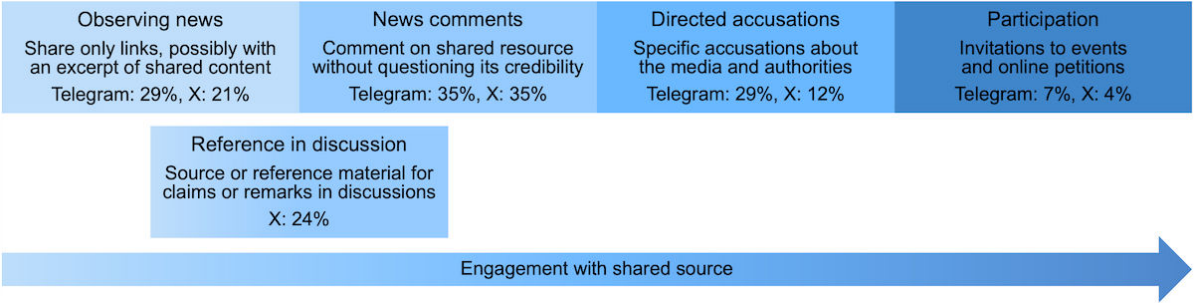
Different themes (rectangular boxes) show the purpose of sharing mainstream information in Telegram and X posts, derived through thematic analysis.

Using thematic analysis (cf Methods), we developed 5 themes describing the purpose of sharing mainstream information in the Telegram and X discourses (Figure 3). The position on the x-axis in Figure 3 indicates the varying engagement with the shared source for the different usage patterns in the examined posts. Adjacent boxes reflect conceptually related themes. The reported percentages in the boxes report the frequency of the themes in the analyzed data. Percentages may not add up to 100 due to rounding errors and uncategorized posts.

#### Observing News

It describes a pattern where only the link to an information source is shared. Sometimes, the shared links are accompanied by an excerpt of the shared source (eg, the title, a quote, or a summary); however, the post does not contain a user comment on the shared content.

#### News Comments

In contrast, news comments describe sharing links along with user-written comments, which refer to the shared information. These comments may be critical and express disapproval of the vaccination and public measures. However, the criticism is not directed at the source and does not explicitly question the source’s credibility. Instead, users may offer personal interpretations, views, or predictions based on the shared information, for instance, on the likelihood of mandatory COVID-19 vaccination. Such comments may also serve to draw attention to specific developments, such as new COVID-19 cases.

#### Directed Accusations

It refers to posts that accuse the media or governmental authorities and their political representatives of inappropriate news coverage or behavior. A key difference from news comments is that links are shared specifically to criticize the source of the information. For news articles, accusations may be made against the publishing media outlet or the individuals or groups featured in the news reports. We observed a wide range of accusations, motivated by different reasoning. This included conspiracy theories (eg, that the pandemic was planned) and the suspicion of bad intentions (eg, the deliberate attempt to limit personal freedom). But reasoning also included the uncoordinated and thus (seemingly) contradictory behavior of policymakers (eg, different implementation of scientific recommendations), the withholding of information in media reporting to avoid addressing the risks of vaccination, and more generally speaking, the neglect of issues that are perceived as important. For X, accusations also involved demands for more strict public measures.

#### Participation

Participation, a fourth theme, describes posts that call for active participation in an event or an online petition. Links are mainly used to share additional information or to register in some way. Events included protests and, on X, also information about available vaccination appointments, while online petitions mainly related to petitions against the introduction of mandatory vaccination.

#### Reference in Discussion

As the last theme, this is exclusive to X. It describes the use of sources for claims or supporting remarks in a thread, that is, as part of a discussion between different users, which is impossible in Telegram channels, as only selected users can post. Examples include news reports on the effectiveness of vaccinations or statistics on case numbers provided online by public authorities.

Finally, we note that it was not always possible to clearly assign each of the analyzed posts to 1 of the 5 themes. In some cases, especially with X posts, the statement made in a post was ambiguous because the context of the posts was unclear. We excluded such posts from the analysis.

## Discussion

### Principal Findings

This study compared the German discourse on the COVID-19 vaccination in critical Telegram communities, known for opposing the COVID-19 vaccination, with the discourse on X (formerly Twitter) and in online news. The study focused on the importance of mainstream information, represented by sources from governmental authorities and large media outlets. More precisely, we analyzed information prevalence, discourse dynamics, and the role of shared information for 5 popular vaccination-related subtopics (death, long COVID, mandatory vaccination, measures in schools, and virus variants). Our results show that mainstream sources were part of the Telegram communities’ information mix, although such sources were shared more often on X. The intensity of the discourse across Telegram, X, and online news showed varying degrees of similarity for the different subtopics, ranging from no association to high positive correlation between week-to-week changes. Mainstream information was shared both as credible information within discussions and, especially on Telegram, for criticizing public health measures and accusing the media and government, indicating an ambivalent relationship.

### Comparison With Prior Work

Overall, we found that opinions on vaccination in the X discourse were fairly balanced between posts from both vaccination advocates and opponents. This impression is consistent with other studies of X posts in the German language that drew similar conclusions regarding the general vaccination discourse [41] and mandatory vaccination in particular [39]. Studies using English X data found similar tendencies, with vaccination mandates emerging as an important topic in the context of the vaccination-critical discourse, aligning with our observations [48,65].

Regarding the Telegram discourse, we found only posts that criticize or reject vaccinations. Again, this agrees with the findings of other studies [20,34]. This one-sidedness was a consequence of the focus on particularly critical Telegram communities.

With regard to the sharing of mainstream information sources, we found that these played a dominant role in vaccination-related discourse on X (Figure 1). This agrees with a study analyzing information sharing in the German vaccination discourse, where 11 out of the 15 most shared links on X were news sources that were included in our study [40]. When it comes to sharing mainstream information on Telegram, the picture is again similar to that found in other studies. The mainstream sources were frequently shared, but the influence of other information sources was relatively greater [20,29,34], especially in comparison with the results for X.

So far, our results are consistent with previous research. However, other studies focused on the analysis of individual platforms (ie, Telegram or X) and, in the case of Telegram, primarily on the analysis of misinformation and disinformation (eg, conspiracy theories). Our study thus provides new insights, particularly with regard to the role of mainstream information in the analyzed Telegram communities. Specifically, in contrast to other studies [29,34], we found that the use of mainstream sources was not limited to expressing rejection of public health measures or trust only in sources that were critical of these measures. Instead, we found an interconnectedness between mainstream information use and platform-specific discussions on Telegram and X that emphasized the need for information during a pandemic that affected daily life.

This can be seen, in particular, for mandatory vaccination. Here, we observed that both posts on Telegram and on X used mainstream information to track developments regarding the potential introduction of mandatory vaccination. In Germany, the debate on general mandatory vaccination gained momentum, particularly from the end of November 2021, after government politicians spoke out in favor of mandatory vaccination in political discussions and interviews with mainstream media (eg, [66,67]). In April 2022, a draft law was put to a vote with the aim of introducing a mandate, which was rejected [68], bringing the debate to an end. This coincides with the period of highest discourse intensity on Telegram, X, and mainstream online news (Figure 2, high correlations between time series).

However, other topics, particularly long COVID, showed a significantly different dynamic and a continuously ambiguous discourse intensity (Figure 2, low correlations between time series). Here, we observed that Telegram posts were particularly critical of mainstream information sources, which, according to these posts, generally did not address long-term consequences associated with vaccination. Posts on X also expressed concerns about long COVID, but also in connection with COVID-19, instead of vaccination effects.

In this context, our findings suggest that persistent disjointed topic dynamics across platforms may be linked to information voids. When information needs (such as concerns about the consequences of vaccination on Telegram) are not being met by the information in mainstream sources, different communities may turn to different alternative information sources, potentially leading to the observed disjointed discourse.

In the case of long COVID, surveys conducted at the end of 2021 and in April 2022 found that around half of respondents said they were poorly informed about long COVID or would like more information [69,70]. Such information voids are problematic because they can promote the spread of misinformation (ie, by filling the gaps) [3,16]. Similar discrepancies can be observed in other (international) contexts. News coverage on vaccination in English-speaking countries increased with the rollout of COVID-19 vaccines from the end of 2020, along with an increased discourse on X/Twitter in the English language, although the discussed vaccination-related topics only partially overlapped [65,71-73].

These findings highlight the complexity of information engagement patterns and suggest that understanding vaccine hesitancy requires examining not just what information is consumed, but how it is interpreted and used within specific community contexts. Combining previous studies and our results, we suggest that different attitudes toward mainstream information sources existed within the examined Telegram communities. Similarly, vaccine acceptance itself is increasingly recognized as a spectrum encompassing a range of attitudes, often varying among individuals in complex and context-specific ways [74,75]. These beliefs can range from apathy to conspiracy theories, and similar attitudes are often clustered in online discourse [75,76], in line with our findings.

Though vaccine-hesitant groups have been associated with lower-quality information online [77], our results suggest that actors from the mainstream information ecosystem can play an active role in shaping opinions in critical online communities. For example, the influence of media on the public discourse on vaccines was especially evident with the European suspension of AstraZeneca’s vaccine. One study suggested that this decision and subsequent news discourse resulted in a decline in vaccine acceptance across Europe [78].

Trust continues to remain the most important predictor of vaccine uptake, meaning that improving attitudes across media relies on balanced reporting in which accurate information promotes both risks and benefits [74,79]. Overall, the goal must be to strengthen these groups’ trust in the information disseminated within the general public debate [74,79]. Critically, the spectrum of hesitant individuals requires differing and tailored strategies to improve overall vaccine acceptance, including strategies, such as tailoring to cultural backgrounds, health literacy levels, or nationality [74,79]. This raises the question of how to consider group-specific information needs within the general public debate while maintaining a balanced reporting that does not jeopardize the general acceptance of vaccination or other public safety measures.

### Limitations and Future Directions

This study has several limitations that may form the basis for further research. First, while we focused on popular vaccination-related topics, the vaccination discourse was complex and included more topics than we investigated. Moreover, we regarded the Telegram communities analytically as a single network, without relating findings to its subcommunities. Future work could, therefore, investigate a broader spectrum of topics in combination with network analysis, which would allow for an even more nuanced breakdown of the relationship of specific Telegram communities to the mainstream discourse [35]. Furthermore, we approximated definitions of mainstream information via source popularity. There are far more media sources than we have included—for instance, in Germany, there are over 300 daily newspapers [80]—and media extends to other digital information sources, such as television. One possibility could be to include national evening television news programs, such as the Tagesschau in Germany [81]. While our selection represents the most popular media sources, a more differentiated examination of the media landscape, in particular how political positions within the mainstream information ecosystem differ, would increase the robustness of the results. Since our study highlighted the benefits of cross-platform analyses, our framework should be extended to other social media platforms to obtain a full picture of the online information behavior, which is essential for the design of effective risk communication. This includes the pathways within social media platforms [14], that is, how information is propagated and amplified within communities, as this study focused mainly on the information flow from mainstream information actors to online communities.

## Conclusions

Our study demonstrates the potential of combining different methods to create a multiperspective analysis, revealing a complex information sharing behavior across platforms with different attitudes toward the COVID-19 vaccination. In the context of infodemics, it is essential to understand the pathways and reach of validated as well as potentially harmful information within an information ecosystem. Demand and supply of information may influence each other; for example, a lack of available information in credible sources (information voids) can favor misinformation. The presented cross-platform approach shows how such potential voids can be detected by comparing discourse patterns across platforms. Our results suggest that critical communities may engage with mainstream discourse in nuanced ways, neither fully rejecting nor accepting mainstream sources, but rather contextualizing them in their own discourse. Consequently, effective risk communication must move beyond simple information dissemination to understand how different communities process and use mainstream information. Future pandemic communication strategies should consider these diverse engagement patterns to ensure that risk communication reaches all parts of the public, building trust through targeted approaches that acknowledge varying information needs and contexts.

## Supplementary material

10.2196/76309 Multimedia Appendix 1 Keyword filtering and text clustering.

10.2196/76309 Multimedia Appendix 2 Selection of mainstream information sources.

10.2196/76309 Multimedia Appendix 3 Time series analysis.

10.2196/76309 Multimedia Appendix 4 Query for retrieving global data on events, location, and tone data.

## Abbreviations

GDELT: Global Data on Events, Location, and Tone
