# Supplementary material for: Information Pathways and Voids in Critical German Online Communities During the COVID-19 Vaccination Discourse: Cross-Platform and Mixed Methods Analysis
Source: J Med Internet Res. 2025 Oct 17;27:e76309. doi: 10.2196/76309 (PMC12557652; doi:10.2196/76309)
Supplement: Multimedia Appendix 1 [file jmir-v27-e76309-s001.pdf]

Multimedia appendix: Keyword filtering and text clustering

In this appendix, we provide further details on the methods described in the main text of the study: code for the initial vaccination-related keyword filtering, the implementation details for the subsequent topic exploration with text clustering, and a description, respectively, code for the second, topic-specific keyword filtering.

Contents

1 Initial content filtering **2**

2 Implementation of the topic exploration **3**

3 Topic filtering **6**

List of Figures

S1 The largest 50 topics for Telegram and X, ordered by size 8

List of Tables

S1 Titles for 10 largest topic cluster for Telegram and X 8

S2 Overlap of posts between topics for Telegram 9

S3 Overlap of posts between topics for X 9

S4 Precision (P) and recall (R) for the classification of text posts and news headlines 10

List of Listings

S1 Python code for classyfing text as vaccination-related 2

S2 Python code used to extract German article headlines from website links 4

S3 Python code for expanding a shortened URL into the original address 5

S4 Python code for the keyword-based multi-topic classifcation 10

1 Initial content filtering

Listing S1 shows the regular expression used to filter vaccination-related texts from the Telegram, X, and news headlines datasets used in the study. It combines a range of vaccine names, vaccine manufacturers as well as words related to ‘Impfung’, respectively, ‘Vakzin’ (vaccine, vaccination) and ‘impfen’, respectively, ‘vakzinieren’ (to vaccinate). We provide a quantitative evaluation of precision and recall for this filter as part of Section 3.

```python
import re

vac_names = [
    'bimervax', 'comirnaty',
    'jcovden', 'nuvaxovid',
    'spikevax', 'sputnik v',
    'vaxzevria',
]

vac_companies = [
    'biontech', 'pfizer',
    'janssen', 'novavax',
    'moderna', 'cansino',
    'curevac', 'johnson & johnson',
    'sinopharm', 'astrazeneca',
]

# ignores lower- and upper case letter
# for stem 'impf', prefixes 'sch' and 'gl' are excluded,
# as including them led to many false positives,
# e.g., German words 'schimpfen' (to scold) or
# 'verunglimpfen' (to disparage sb)
vac_combined = f'{"|".join(vac_names)}|{"|".join(vac_companies)}'
impf_regex = re.compile(
    rf'(((?<!sch)(?<!gl)impf[a-z\-]*)|vakzin[a-z\-]*|{vac_combined})',
    re.IGNORECASE
)

def is_vacc(text: str) -> bool:
    """This function classifies a text as vaccination-related (or not)."""
    return impf_regex.search(text) is not None
```

Listing S1: Python code for classyfing text as vaccination-related.

2 Implementation of the topic exploration

Here, we describe the technical implementation of the topic exploration. We used the Telegram and X datasets described in the main text that had already been filtered to only contain vaccination-related content (cf. Section 1).

Preprocessing

We performed minimal text preprocessing for the following text clustering. First, we removed user mentions, which we had previously replaced with a placeholder token ('@user'). Second, instead of simply removing or replacing links with a placeholder token, as it is common (1, 2), we extracted headlines from links, possibly providing additional information to the clustering process. More precisely, we simply extracted the last part of the path from URL links, removed separators between words (such as '-') and used fastText (3) to ensure that the resulting text represented German language. We removed the links within the texts that were not classified as German, as well as links pointing to social media platforms. We provide sample code in Listing S2. We note that all of the website links embedded in the X dataset, and in some cases in the Telegram dataset, where shortened links, and thus hid the final location. Therefore, we first expanded these shortened links before extracting. We provide sample code in Listing S3.

Clustering framework

We used the BERTopic framework (4) for text clustering. BERTopic represents texts as embeddings, which serve as basis for the clustering. We used the *German BERT large paraphrase cosine* model (5) to transform X and Telegram posts into embeddings as this models has proven useful for text clustering (2). We mainly used BERTopic's standard pipeline and model parameters, i.e., we first reduced the dimensionality of the post embeddings to five dimensions using UMAP (6, 7) and then clustered the lower-dimensional representations using HDBSCAN (8). The only deviation from the standard values of the BERTopic pipeline was an increase in the minimum cluster size for HDBSCAN from 15 to 30 in order to prevent the formation of very small clusters.

This resulted in 2243 topic clusters for the Telegram dataset and 6181 topic clusters for the X/Twitter dataset. This initial result contained a large number of outliers for both datasets (up to 50% of the datasets). Thus, we used BERTopic's built-in functionality to automatically reduce outliers (9) (using the embeddings strategy with a threshold value of 0.70). This reduced the outlier for both datasets to around 30%. We ensured clustering quality by manually inspecting clusters, whereby posts from individual clusters must predominantly reveal a common topic. These computations were run on a local cluster in a multi-CPU setup with a single NVIDIA A100 GPU.

Title generation

We used the large language model Mixtral 8x7B (10) to generate short titles for topics in addition to the descriptive keywords extracted through BERTopic (similar to Kloo et al. (11)). We used the following prompt:

```
Write a short title for the following documents: [DOCUMENTS]. Keep it short and reply
with one title.
```

We replaced `[DOCUMENTS]` with 10 representative posts, i.e., posts whose embeddings are the most similar to the average of all post embeddings in a topic (as measured by cosine similarity). We ran Mixtral 8x7B on a local cluster using the Transformers framework (12) and 4 NVIDIA A100 GPUs.

```
import os
import re
from urllib.parse import unquote, urlparse

import fasttext


# replace with actual path
lang_model = fasttext.load_model('path_to_fasttext_model')
)

def get_title(url: str) -> str:
    """Extract article title from last part of url link."""
    if url == '':
        return 'none'

    try:
        res = urlparse(url)
    except:
        print(f'had to correct: {url}')
        url = url.replace('ü', 'oe').replace('ä', 'ae').replace('ö', 'oe')
        res = urlparse(url)

    netloc = res.netloc.lower().split('www.')[-1]
    for excluded_domain in [
        't.me', 't.co', 'twitter.com', 'facebook.com', 'youtube.com',
        'youtu.be', 'vimeo.com', 'tiktok.com', 'instagram.com',
    ]:
      if excluded_domain == netloc:
        return 'none'

    title = unquote(res.path)
    title = title.split('.')[0]
    title = title.strip('/')
    title = title.split('/')[-1]
    title = title.replace('-', ' ').replace('_', ' ')
    title = title.lstrip('0123456789').rstrip('0123456789')

    if lang_model.predict(title)[0][0] == '__label__de':
        return title
    else:
        return 'none'
```

Listing S2: Python code used to extract German article headlines from website links.

```python
import os
import requests
import sys
import re
import tldextract


def is_short_url(url: str) -> bool:
    """Check if a given url is a short url."""
    components = tldextract.extract(url.lower())
    return components.domain + '.' + components.suffix in [
        'bit.ly', 'is.gd', 'dlvr.it', 'tinyurl.com', 't.co',
    ]

def get_expanded_url(url: str, levels=3) -> str:
    """Expand a shortened url by following the HTTP header's location."""
    try:
        for _ in range(levels):
            response = requests.head(re.sub(r'[\.:;,\-]+$', '', url))
            url = response.headers['location']

            if not is_short_url(url):
                break
        return url

    except Exception as e:
        print(f'url: {url} failed', file=sys.stderr)
        return ''
```

Listing S3: Python code for expanding a shortened URL into the original address.

3 Topic filtering

We selected a subset of vaccination-related topics for detailed quantitative and qualitative analysis.

Platform-specific clustering

We based the selection of topics on the outcome of the text clustering, focusing on the 10 most popular topics for Telegram and X, i.e., topics with the most posts (cf. Figure S1 and Table S1).

Initially, we experimented with clustering the combined datasets of Telegram and X. However, the resulting clusters were dominated by X content due to the larger size of the X dataset, which biased the clustering toward X's dominant topics. Instead, we clustered the dataset independently, allowing us to investigate topics separately for both platforms. This approach yielded partially different topic sets for each platform, limiting direct cross-platform comparability. Thus, we developed a keyword-based classification scheme, as described in the following subsection. We note that probabilistic topic modeling could have served as an alternative to text clustering (13). Yet, text clustering may work better for short texts such as X posts (14). Both techniques, topic modeling and text clustering, have been successfully used for social media text analysis (e.g., (2, 15)), and should be chosen based on the use case. Initial experiments confirmed that text clustering was more suitable for the exploration of dominant topics in our predominantly short-text datasets.

Keyword-based topic definition

To ensure consistent topic definitions across platforms, we created keyword-based topic categories. We then applied these keywords as a second-level filter to all datasets (Telegram, X, news headlines), building on the initial vaccination-related content filtering. We note that this is a similar approach to Purnat et al. (2021), who developed a multi-level, keyword-based taxonomy to classify posts from different social media platforms related to the COVID-19 pandemic. This approach made it straightforward to assign multiple topics to individual texts. During the initial topic exploration, we had observed that especially Telegram posts often dealt with multiple topics (cf. topic overlaps for Telegram and X in Tables S2 and S3). We based our keyword selection on the output of the BERTopic clustering pipeline. This pipeline uses a statistical process based on the *term frequency–inverse document frequency* measure, which determines the relevance of words in a text collection, to generate descriptive keywords for each topic cluster. Additionally, we manually analyzed random post samples from each topic to identify the most precise keywords with clear topical relevance. The code used to classify social media posts and news headlines is shown in Listing S4.

Method validation

We evaluated the classification quality by calculating precision and recall metrics. For each topic in each dataset, we analyzed random samples of 50 texts classified as topic-relevant and 50 classified as non-relevant (Table S4), totaling 1,800 samples. We calculated precision and precision as follows

$$precision = \frac{TP}{TP+FP},$$
$$recall = \frac{TP}{TP+FN},$$

where TP denotes the number of true positives (correctly classified samples), FP the number of false positives (samples incorrectly classified as belonging to a topic), and FN denotes the number of false negatives (samples incorrectly classified as not belonging to a topic). We classified a sample as a false positive when the identified keyword was either not related to the classified topic or the text discussed the classified topic in a context different from vaccination. For samples related to the first-level filter (vaccination-related content), we included samples

that were classified as vaccination and those that were classified as not related to vaccination. For samples related to the second-level filter, we only considered samples that were already classified as related to vaccination.

First-level vaccination content filtering achieved perfect recall (1.0) across all datasets. Second-level topic filtering maintained perfect recall for four of five subtopics across all platforms. For first-level filtering, the precision was also 1.0. For the subsequent filtering of vaccination-related topics, the precision was generally lower, but apart from one case, it was always above 0.9. We observed that texts were sometimes misclassified because keywords had a different meaning in the context of the post or news headline. For example, in one case, a text containing the word 'Musterschüler' (model student) was assigned to the topic measures in schools; however, this post was about the assessment of a country's performance in coping with the pandemic. Despite occasional misclassifications, our keyword filtering approach proved reliable and consistent with established social media research methodologies (2, 16).

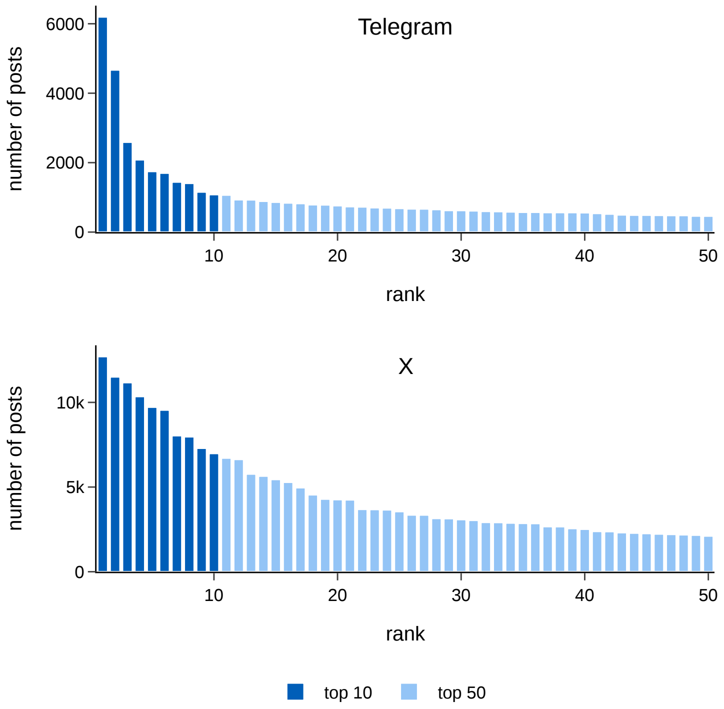

Figure S1: The largest 50 topics for Telegram and X, ordered by size (rank).

Rank	Telegram	X
1	Documented Cases of Adverse Reactions and Deaths Following COVID- 19 Vaccination	Divided Opinions: Sputnik V's Global Adoption and Criticism
2	Impfstoff and Related Topics	Debating the Effectiveness of Current and Omicron-Specific Vaccines Amid Rising Concerns
3	Corona Vaccine Emergency Approval, Söder Backs Vaccine Mandate, and Vaccine Misinformation	Critical Analysis of Covid-19 and Vaccination: Weighing the Risks and Long- Term Consequences
4	Healthcare Workers' Protest: Free Vaccination Choice & Abolition of 'Einrichtungsbezogene Impfpflicht'; Düsseldorf Rally for Immediate Restoration of All Fundamental Rights	Debating the Effectiveness and Risks of Corona Vaccinations: A Collection of Contrasting Views
5	Bill Gates: Philanthrop or Greedy Pandemic Profiteer? Examining His Power and Motives	Urgent Call for School Immunization: Protecting Students and Their Families
6	Israel's Shift in COVID-19 Strategy: High Infection Rates and Waning Confidence in Vaccines	Impact of Vaccination on Delta and Other COVID-19 Variants: An Overview of Current Research
7	Germany: School Imposes Increased Pressure for Vaccination	Questioning the Effectiveness and Necessity of Frequent Booster Shots and Mandatory Vaccination
8	Österreich Suspends Coronavirus Vaccine Mandate Amid Growing Opposition	Impfpflicht: Necessity for Preventing Future COVID-19 Waves
9	Putin to Get Russian-Developed COVID-19 Vaccine 'Sputnik V'	Local COVID-19 Vaccination Opportunities: Walk-in Clinics & Mobile Teams
10	Reevaluating the Effectiveness and Implications of COVID-19 Vaccines	Attitudes Towards Vaccination and Mask-Wearing Amidst Pandemic

Table S1: Titles for 10 largest topic cluster for Telegram and X. Note: Titles were generated using Mixtral 8x7B.

Topic		Death	Long COVID	Mandatory vaccination	Measures in schools	Virus variants
Death	(*n=61,956*)	100.0%	1.6%	12.8%	2.9%	2.6%
Long COVID	(*n=4,017*)	24.4%	100.0%	13.0%	4.9%	2.3%
Mandatory vaccination	(*n=88,312*)	9.0%	0.6%	100.0%	4.3%	2.4%
Measures in schools	(*n=13,427*)	13.5%	1.5%	28.5%	100.0%	2.1%
Virus variants	(*n=9,242*)	17.3%	1.0%	22.8%	3.0%	100.0%

Table S2: Overlap of posts between topics for Telegram. Note: Numbers represent overlap of posts between topics as percentages, relative to topic shown in the row.

Topic		Death	Long COVID	Mandatory vaccination	Measures in schools	Virus variants
Death	(*n=116,376*)	100.0%	2.4%	8.0%	0.9%	2.2%
Long COVID	(*n=26,106*)	10.6%	100.0%	5.2%	3.2%	3.2%
Mandatory vaccination	(*n=245,826*)	3.8%	0.6%	100.0%	1.4%	2.1%
Measures in schools	(*n=40,369*)	2.5%	2.0%	8.6%	100.0%	2.6%
Virus variants	(*n=43,748*)	5.8%	1.9%	11.9%	2.4%	100.0%

Table S3: Overlap of posts between topics for X. Note: Numbers represent overlap of posts between topics as percentages, relative to topic shown in the row.

```python
import re


TAGS = {
    'school': [
        'schule', 'schüler', 'lehrer', 'lehrperson'
        ],
    'variants': [
        r'#?alpha(\-[a-z]*|\b)', 'delta', 'omikron'
        ],
    'long_covid': [
        r'long[\- ]?covid', r'post[\- ]?covid', r'post[\- ]?vac',
        'langzeitfolgen?', 'langzeitsch[aä]den'
        ],
    'mandatory_vacc': [
        'zwang', 'freiheit', 'impfpflicht'
        ],
    'death': [
        'tod', r'tot\b|tote|toter|toten', 'gestorben', 'starb',
        'sterbe', 'stirbt', 'stirbst', 'sterbt', 'sterben'
        ],
}

def tag_post(post: str) -> dict[str, bool]:
    """This function classifies a post based on 'regex-enhanced' keyword
    filtering."""
    result = {}

    # '(?i) ignores lower- and upper-case letters'
    for topic, tagset in TAGS.items():
        if re.search(
            f"(?i){'|'.join(tagset)}",
            post,
        ) is not None:
            result[topic] = True
        else:
            result[topic] = False

    return result
```

Listing S4: Python code for the keyword-based multi-topic classifcation.

Datasets	Telegram P	Telegram R	X P	X R	News headlines P	News headlines R
Vaccination	1.00	1.00	1.00	1.00	1.00	1.00
Death	0.96	0.96	1.00	1.00	0.92	1.00
Long COVID	0.98	1.00	1.00	1.00	1.00	1.00
Mandatory vaccination	0.98	1.00	0.96	0.98	1.00	1.00
Measures in school	0.88	1.00	0.94	1.00	1.00	0.98
Virus variants	0.98	1.00	1.00	1.00	1.00	1.00

Table S4: Precision (P) and Recall (R) for the classification of text posts and news headlines. Note: Measures were calculated using a random sample of 50 included and 50 excluded tests per dataset and topic.

References

[1] Arnaldo Santoro, Alessandro Galeazzi, Teresa Scantamburlo, Andrea Baronchelli, Walter Quattrociocchi, and Fabiana Zollo. Analyzing the changing landscape of the covid-19 vaccine debate on twitter. *Social Network Analysis and Mining*, 13(1):115, Sep 2023. doi: 10.1007/s13278-023-01127-3.

[2] Silvan Wehrli, Chisom Ezekannagha, Georges Hattab, Tamara Boender, Bert Arnrich, and Christopher Irgang. Guiding sentiment analysis with hierarchical text clustering: Analyzing the German X/Twitter discourse on face masks in the 2020 COVID-19 pandemic. In Orphée De Clercq, Valentin Barriere, Jeremy Barnes, Roman Klinger, João Sedoc, and Shabnam Tafreshi, editors, *Proceedings of the 14th Workshop on Computational Approaches to Subjectivity, Sentiment, & Social Media Analysis*, pages 153–167, Bangkok, Thailand, August 2024. Association for Computational Linguistics. doi: 10.18653/v1/2024.wassa-1.13.

[3] Armand Joulin, Edouard Grave, Piotr Bojanowski, and Tomas Mikolov. Bag of tricks for efficient text classification. In Mirella Lapata, Phil Blunsom, and Alexander Koller, editors, *Proceedings of the 15th Conference of the European Chapter of the Association for Computational Linguistics: Volume 2, Short Papers*, pages 427–431, Valencia, Spain, April 2017. Association for Computational Linguistics. URL `https://aclanthology.org/E17-2068`.

[4] Maarten Grootendorst. Bertopic: Neural topic modeling with a class-based tf-idf procedure. *arXiv preprint arxiv:2203.05794*, 2022.

[5] Phillip May, Deutsche Telekom AG, and deepset GmbH. German BERT large paraphrase cosine. `https://huggingface.co/deutsche-telekom/gbert-large-paraphrase-cosine`, 2023. accessed 2024-05-13.

[6] Leland McInnes, John Healy, Nathaniel Saul, and Lukas Großberger. Umap: Uniform manifold approximation and projection. *Journal of Open Source Software*, 3(29):861, 2018. doi: 10.21105/joss.00861.

[7] Sebastian Raschka, Joshua Patterson, and Corey Nolet. Machine learning in python: Main developments and technology trends in data science, machine learning, and artificial intelligence, 2020. URL `https://arxiv.org/abs/2002.04803`.

[8] Leland McInnes, John Healy, and Steve Astels. hdbscan: Hierarchical density based clustering. *Journal of Open Source Software*, 2(11):205, 2017. doi: 10.21105/joss.00205.

[9] Maarten Grootendorst. Outlier reduction. `https://maartengr.github.io/BERTopic/getting_started/outlier_reduction/outlier_reduction.html#embeddings`, 2024. accessed 2025 Jan 15.

[10] Albert Q. Jiang, Alexandre Sablayrolles, Antoine Roux, Arthur Mensch, Blanche Savary, Chris Bamford, Devendra Singh Chaplot, Diego de las Casas, Emma Bou Hanna, Florian Bressand, Gianna Lengyel, Guillaume Bour, Guillaume Lample, Lélio Renard Lavaud, Lucile Saulnier, Marie-Anne Lachaux, Pierre Stock, Sandeep Subramanian, Sophia Yang, Szymon Antoniak, Teven Le Scao, Théophile Gervet, Thibaut Lavril, Thomas Wang, Timothée Lacroix, and William El Sayed. Mixtral of experts. *arXiv preprint arxiv:2401.04088*, 2024.

[11] Ian Kloo, Iain J. Cruickshank, and Kathleen M. Carley. A cross-platform topic analysis of the nazi narrative on twitter and telegram during the 2022 russian invasion of ukraine. *Proceedings of the International AAAI Conference on Web and Social Media*, 18(1):839–850, May 2024. doi: 10.1609/icwsm.v18i1.31356.

[12] Thomas Wolf, Lysandre Debut, Victor Sanh, Julien Chaumond, Clement Delangue, Anthony Moi, Pierric Cistac, Tim Rault, Rémi Louf, Morgan Funtowicz, Joe Davison, Sam Shleifer, Patrick von Platen, Clara Ma, Yacine Jernite, Julien Plu, Canwen Xu, Teven Le Scao, Sylvain Gugger, Mariama Drame, Quentin Lhoest, and Alexander M. Rush. Huggingface's transformers: State-of-the-art natural language processing, 2020. URL `https://arxiv.org/abs/1910.03771`.

[13] David M. Blei. Probabilistic topic models. *Commun. ACM*, 55(4):77–84, apr 2012. ISSN 0001-0782. doi: 10.1145/2133806.2133826.

[14] Roman Egger and Joanne Yu. A topic modeling comparison between LDA, NMF, Top2Vec, and BERTopic to demystify Twitter posts. *Frontiers in Sociology*, 7, 2022. doi: 10.3389/fsoc.2022.886498.

[15] Maximilian Zehring and Emese Domahidi. German corona protest mobilizers on telegram and their relations to the far right: A network and topic analysis. *Social Media + Society*, 9(1):20563051231155106, 2023. doi: 10.1177/20563051231155106.

[16] Tina D Purnat, Paolo Vacca, Christine Czerniak, Sarah Ball, Stefano Burzo, Tim Zecchin, Amy Wright, Supriya Bezbaruah, Faizza Tanggol, Ève Dubé, Fabienne Labbé, Maude Dionne, Jaya Lamichhane, Avichal Mahajan, Sylvie Briand, and Tim Nguyen. Infodemic signal detection during the covid-19 pandemic: Development of a methodology for identifying potential information voids in online conversations. *JMIR Infodemiology*, 1(1):e30971, 2021. ISSN 2564-1891. doi: 10.2196/30971.
