# Supplementary material for: Information Pathways and Voids in Critical German Online Communities During the COVID-19 Vaccination Discourse: Cross-Platform and Mixed Methods Analysis
Source: J Med Internet Res. 2025 Oct 17;27:e76309. doi: 10.2196/76309 (PMC12557652; doi:10.2196/76309)
Supplement: Multimedia Appendix 2 [file jmir-v27-e76309-s002.pdf]

Multimedia appendix: Selection of mainstream information sources

In this section, we describe the approach to selecting the mainstream information sources used in the study.

Contents

1 Concept **2**

2 Technical implementation **2**

3 Additional results **2**

List of Figures

S1 The 10 most frequently shared domains per category of mainstream information source considered in the study (private media, public media, and governmental authorities) 5

S2 The number of links shared with respect to the top-level domains of the links for Telegram and X posts 5

List of Tables

S1 Websites of mainstream information sources used for the analyses 3

List of Listings

S1 Python code used to extract domains from texts 4

1 Concept

For the analysis of mainstream information, we used sources from government authorities and private and public media (see Table S1 for a detailed overview). We included sources from Germany, Austria, and Switzerland. For sources related to the government, we took into account authorities at both the national and state level. More precisely, we included the websites of the national government, national ministries, national public health institutes, national statistics offices, and websites of all federal states (for Switzerland, those of the cantons). We note that the number of included websites varied from country to country due to the different number of federal states, respectively, cantons and the different uses of shared domains. For example, many Austrian federal states use the same domain ('gv.at') but with different subdomains, while each Swiss canton has its own domain. Finally, we included the website of the *World Health Organization* as the only supranational organization, given its central role during the COVID-19 pandemic.

For the selection of media sources, we focused on the most popular online sources, i.e., those with the widest reach. We differentiated between private and public media. The former included the online news websites of privately owned and financed media outlets, and the latter were those of publicly financed and owned media organizations (ARD in Germany, ORF in Austria, and SRG SSR in Switzerland). We used the *monthly unique user* as a selection criterion, which measures the number of users who visited a particular website at least once in one month. As a starting point, we considered all websites that reached at least 10% of the population. We chose this threshold empirically because it includes the web portals of Germany's largest national daily newspapers. For the population values, we considered the values for 2023, although, for Switzerland, we only considered the share of the German-speaking population (1–4). We then cross-referenced the initial selection of websites with the results of the *Reuters Institute Digital News Report* for 2019–2023 (5–9), which uses surveys in different countries each year to determine the most popular online media offerings. The survey results were used to validate the initial selection and to include other popular online news services for which no usage data was available (e.g., msn.com). The reports also had the advantage of being available for all years of the study's analysis period. In contrast, the numbers for monthly unique users for the various media services were sometimes only available at different points within the analyzed period. The surveys only provided aggregated results for the different German public broadcasters' online news services, for which the number of unique monthly users was also not published. For these services, we used the monthly number of visits. We included websites with at least as many monthly views as the smallest media portal from a German private media outlet, i.e., with the fewest monthly unique users. All data used for the selection of media websites is referenced in Table S1.

2 Technical implementation

To analyze the usage of mainstream online sources in the analyzed Telegram and X posts, we used the Python *URLExtract* library (10) to first extract website links from the post texts, and then determined the domain of these links with the Python *tldextract* library (11). We provide sample code in Listing S1.

3 Additional results

Fig S2 and Fig S1 provide additional results on the prevalence of the analyzed websites, i.e., with respect to the top-level domain (e.g., .de) and the 10 most frequently shared domains. These results refer to posts assigned to one of the five subtopics analyzed in the study.

Category	Country	Domains
Governmental authorities	Germany	auswaertiges-amt.de, baden-wuerttemberg.de, bayern.de, berlin.de, bmas.de, bmbf.de, bmelv.de, bmfsfj.de, bmj.de, bmu.de, bmvg.de, bmvi.de, bmwk.de, bmz.de, brandenburg.de, bund.de, bundesfinanzministerium.de, bundesgesundheitsministerium.de, bundesregierung.de, bundestag.de, destatis.de, hamburg.de, hessen.de, land.nrw, landesportal.bremen.de, niedersachsen.de, regierung-mv.de, rki.de, rlp.de, saarland.de, sachsen-anhalt.de, sachsen.de, schleswig-holstein.de, thueringen.de
	Austria	burgenland.at, gv.at, sozialministerium.at, statistik.at, verwaltung.steiermark.at, voralberg.at
	Switzerland	admin.ch, ag.ch, ai.ch, ar.ch, bern.ch, bs.ch, baselland.ch, fr.ch, gl.ch, gr.ch, lu.ch, nw.ch, ow.ch, sg.ch, sh.ch, so.ch, sz.ch, tg.ch, ur.ch, vs.ch, zug.ch, zh.ch
	Int. Organization	who.int
Public media	Germany[1]	br.de (12), dw.com (13), heute.de (12), mdr.de (12), ndr.de (12), swr.de (12), tagesschau.de (12), wdr.de (12), zdf.de (12)
	Austria	orf.at (14)
	Switzerland	srf.ch (9), teletext.ch (15)
Private media	Germany	bild.de (16), faz.net (17), focus.de (17), gmx.de[2] (18), msn.de[2] (9), n-tv.de (19), n24.de (20), rnd.de (17), spiegel.de (17), stern.de (21), sueddeutsche.de (17), t-online.de (22), tagesspiegel.de (17), web.de (23), welt.de (20), zeit.de (17)
	Austria	derstandard.at (14), diepresse.com (14), exxpress.at (14), gmx.at[2] (14), heute.at (14), kleinezeitung.at (14), krone.at (14), kurier.at (14), meinbezirk.at (14), msn.at[2] (9), nachrichten.at (14), noen.at (14), oe24.at (14), salzburg24.at (14), sn.at (14), tt.com (14), vienna.at (14), vol.at (14)
	Switzerland	20min.ch (24), aargauerzeitung.ch (25), blick.ch (26), bluewin.ch (27), gmx.ch[2] (9), luzernerzeitung.ch (28), msn.ch[2] (9), nau.ch (29), nzz.ch (30), tagesanzeiger.ch (31), watson.ch (32)

[1]For these websites, we used the number of monthly website visits, which we compared to the website views of included private media outlets in Germany (33).
[2]For these websites, we also considered the country-unspecific top-level domains, i.e., gmx.net and msn.com.

Table S1: Websites of mainstream information sources used for the analyses. Note: Websites are ordered alphabetically. For private and public media, the table contains the sources used as a criterion for their selection. Unless otherwise stated, the references provide information on the number of monthly users or survey results on popular online news media.

```python
import tldextract
from urlextract import URLExtract


from extractor = URLExtract()
# >> extractor.find_urls << extracts finds all URLS in a given text


def extract_domain(u: str) -> str:
    """Extract domain from a given url."""
    extracted = tldextract.extract(u)
    # exception as correct extraction failed otherwise
    if extracted.suffix == 'gv.at':
        return extracted.suffix

    return extracted.domain + '.' + extracted.suffix
```

Listing S1: Python code used to extract domains from texts.

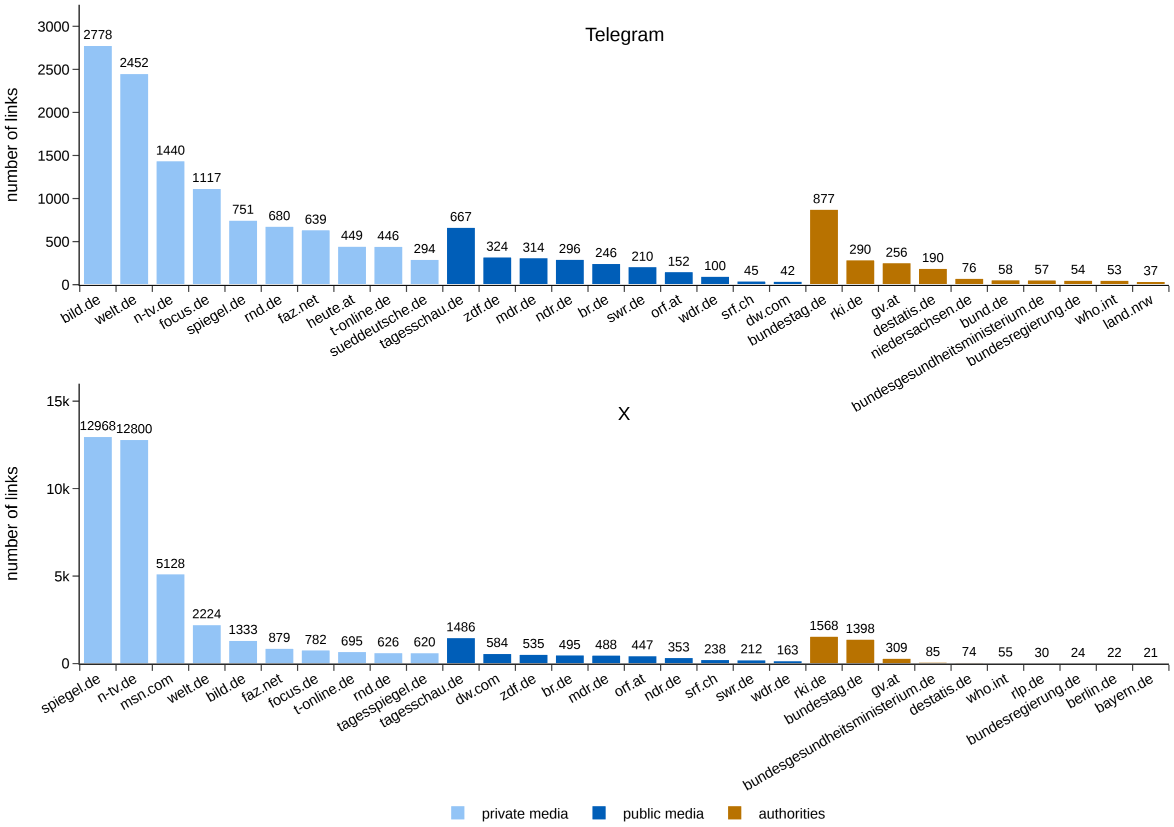

Figure S1: The 10 most frequently shared domains per category of mainstream information source considered in the study (private media, public media, and governmental authorities). The numbers only include posts assigned to one of the analyzed subtopics *death*, *long COVID*, *measures in schools*, *mandatory vaccination*, or *virus variants*.

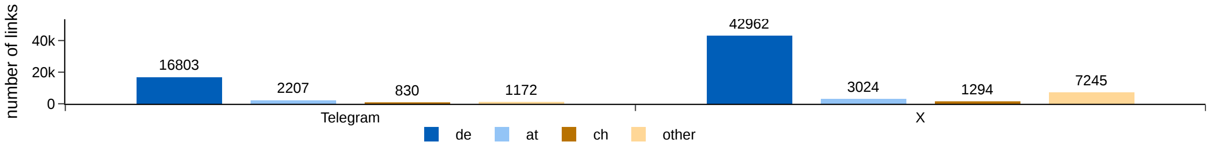

Figure S2: The number of links shared with respect to the top-level domains of the links for Telegram and X posts. The numbers only include posts assigned to the subtopics of *death*, *long COVID*, *measures in schools*, *mandatory vaccination*, or *virus variants*.

References

[1] Presence Switzerland. Language. https://www.eda.admin.ch/aboutswitzerland/en/home/gesellschaft/sprachen.html, 2024. accessed 2025 Jan 14.

[2] Bundesamt für Statistik. Ständige Wohnbevölkerung der Schweiz von 2005 bis 2023. https://de.statista.com/statistik/daten/studie/19317/umfrage/gesamtbevoelkerung-in-der-schweiz, 2024. accessed 2025 Jan 14.

[3] Statistik Austria. Bevölkerung von Österreich von 2014 bis 2024. https://de.statista.com/statistik/daten/studie/19292/umfrage/gesamtbevoelkerung-in-oesterreich, 2024. accessed 2025 Jan 14.

[4] Statistisches Bundesamt. Entwicklung der Gesamtbevölkerung Deutschlands von 1871 bis 2023. https://de.statista.com/statistik/daten/studie/1358/umfrage/entwicklung-der-gesamtbevoelkerung-deutschlands, 2024. accessed 2025 Jan 14.

[5] Nic Newman, Richard Fletcher, Antonis Kalogeropoulos, and Rasmus Kleis Nielsen. Reuters Institute Digital News Report 2019. https://doi.org/10.60625/risj-qzg6-ge29, 2020. accessed 2025 Feb 02.

[6] Nic Newman, Richard Fletcher, Anne Schulz, Simge Andı, and Rasmus Kleis Nielsen. Reuters Institute Digital News Report 2020. https://doi.org/10.60625/risj-048n-ap07, 2020. accessed 2025 Jan 13.

[7] Nic Newman, Richard Fletcher, Anne Schulz, Simge Andı, Craig T. Robertson, and Rasmus Kleis Nielsen. Reuters Institute Digital News Report 2021. https://doi.org/10.60625/risj-7khr-zj06, 2021. accessed 2025 Jan 13.

[8] Nic Newman, Richard Fletcher, Kirsten Eddy, Craig T. Robertson, and Rasmus Kleis Nielsen. Reuters Institute Digital News Report 2022. https://doi.org/10.60625/risj-x1gn-m549, 2022. accessed 2025 Jan 13.

[9] Nic Newman, Richard Fletcher, Kirsten Eddy, Craig T. Robertson, and Rasmus Kleis Nielsen. Reuters Institute Digital News Report 2023. https://doi.org/10.60625/risj-p6es-hb13, 2023. accessed 2025 Jan 13.

[10] lipoja. URLExtract (Version 1.9)). GitHub, 2024. https://github.com/lipoja/URLExtract.

[11] john kurkowski. tldextract (Version 5.1.2)). GitHub, 2024. https://github.com/john-kurkowski/tldextract.

[12] ARD. Online-Nutzung: ARD-Reichweitedaten. https://www.ard.de/die-ard/aufgaben-der-ard/Onlinenutzung-100/, 2023. accessed 2024 Sep 27.

[13] Deutsche Welle. Deutsche Welle: Nutzungszahlen steigen 2023 deutlich. https://corporate.dw.com/de/deutsche-welle-nutzungszahlen-steigen-2023-deutlich/a-67214177, 2023. accessed 2025 Jan 13.

[14] ÖWA. ÖWA Kennzahlen. https://oewa.at/ausweisung, 2023. accessed 2025 Jan 13.

[15] SRG SSR. Der Teletext heute. https://www.srgssr.ch/de/news-medien/dossiers/digitalisierung/wo-will-die-srg-hin-1, 2024. accessed 2025 Jan 13.

[16] agof. Anzahl der Unique User von Bild.de von September 2022 bis Dezember 2022. https://de.statista.com/statistik/daten/studie/303515/umfrage/besucher-von-bildde, 2023. accessed 2025 Jan 13.

[17] agma. Reichweite der Top-10-Nachrichtenseiten in Deutschland im März 2023. https://de.statista.com/statistik/daten/studie/165258/umfrage/reichweite-der-meistbesuchten-nachrichtenwebsites, 2023. accessed 2025 Jan 13.

[18] agma. Anzahl der Unique User von GMX von Februar 2022 bis März 2023. https://de.statista.com/statistik/daten/studie/417955/umfrage/online-besucherzahlen-von-gmx-als-zeitreihe, 2023. accessed 2025 Jan 13.

[19] agof. Anzahl der Unique User von n-tv.de von März 2022 bis Juni 2022. https://de.statista.com/statistik/daten/studie/163609/umfrage/online-besucherzahlen-von-ntv-als-zeitreihe, 2022. accessed 2025 Jan 13.

[20] agof. Anzahl der Unique User von Welt.de von September 2022 bis Dezember 2022. https://de.statista.com/statistik/daten/studie/382245/umfrage/besucher-von-faznet, 2023. accessed 2025 Jan 13.

[21] agof. Anzahl der Unique User von stern.de von September 2022 bis Dezember 2022. https://de.statista.com/statistik/daten/studie/327311/umfrage/besucher-von-sternde/e, 2023. accessed 2025 Jan 13.

[22] agof. Anzahl der Unique User von T-Online.de von Mai 2018 bis November 2021. https://de.statista.com/statistik/daten/studie/417227/umfrage/online-besucherzahlen-von-tonline-als-zeitreihe, 2022. accessed 2025 Jan 13.

[23] agma. Anzahl der Unique User von Web.de von Februar 2022 bis März 2023. https://de.statista.com/statistik/daten/studie/417251/umfrage/online-besucherzahlen-von-web-de-als-zeitreihe, 2023. accessed 2025 Jan 13.

[24] Goldbach. Mediadaten von 20 Minuten. https://goldbach.com/ch/de/kmu-loesungen/20-minuten/profil, 2024. accessed 2024 Sep 27.

[25] CH Media Werbung. # Kennzahlen. https://chmediawerbung.ch/de/marken/aargauer-zeitung-aargauerzeitung-ch, 2024. accessed 2024 Sep 27.

[26] Ringier Advertising. Media Facts. https://www.ringier-advertising.ch/de/brand/blick-ch/#media-facts, 2024. accessed 2024 Sep 27.

[27] Ringier Advertising. Media Facts. https://www.ringier-advertising.ch/de/brand/blue-news/#acc-media-facts, 2024. accessed 2024 Sep 27.

[28] CH Media Werbung. # Kennzahlen. https://chmediawerbung.ch/de/marken/luzerner-zeitung-luzernerzeitung-ch, 2024. accessed 2024 Sep 27.

[29] Nau.ch. Über uns. https://nau-media.ch, 2024. accessed 2024 Sep 27.

[30] NZZone. Mediadaten. https://www.nzzone.ch/produkte/nzz-ch, 2024. accessed 2024 Sep 27.

[31] Goldbach. Mediadaten von Tages-Anzeiger. https://goldbach.com/ch/de/kmu-loesungen/tages-anzeiger/profil, 2024. accessed 2024 Sep 27.

[32] CH Media Werbung. # Kennzahlen. https://chmediawerbung.ch/de/marken/watson, 2024. accessed 2024 Sep 27.

[33] IVW Switzerland. IVW: Anzahl der Visits ausgewählter Nachrichtenportale regionaler Zeitungsangebote in Deutschland im Mai 2024. https://de.statista.com/statistik/daten/studie/1087098/umfrage/anzahl-der-visits-von-nachrichtenportalen-regionaler-zeitungen, 2024. accessed 2025 Jan 14.
