# Supplementary material for: Information Pathways and Voids in Critical German Online Communities During the COVID-19 Vaccination Discourse: Cross-Platform and Mixed Methods Analysis
Source: J Med Internet Res. 2025 Oct 17;27:e76309. doi: 10.2196/76309 (PMC12557652; doi:10.2196/76309)
Supplement: Multimedia Appendix 3 [file jmir-v27-e76309-s003.pdf]

Multimedia appendix: Times series analysis

We provide the full results of the time series analysis presented in the main text: Table S1 shows detailed Pearson correlation coefficients for the temporal relationships considered in the study.

	cross-dataset (all data)			intra-platform (all data, subset)	
Topic	Telegram, X	Telegram, news	X, news	Telegram	X
Death	0.36	0.29	0.36	0.49	0.71
Long COVID	0.29	0.13	0.03	0.33	0.59
Mandatory vaccination	0.88	0.64	0.65	0.76	0.85
Measures in schools	0.43	0.33	0.56	0.53	0.72
Virus variants	0.70	0.40	0.62	0.80	0.76

Table S1: Pearson correlation coefficients between Telegram, X, and mainstream news headlines datasets, considering full time series between April 2019 and February 2023. Note: The datasets consist of the number of weekly posts per topic, with first-order differences used to calculate the coefficients. 'All data' includes all posts for a topic within a dataset, whereas 'subset' only includes posts with at least one link to governmental authorities, public, or private media outlets.

We note that the time series begin in April 2019, i.e., before the first detected COVID-19 cases (end of 2019 (1)). While the study focuses specifically on the COVID-19 vaccination, our approach did not exclude the potential discourse on vaccinations against other diseases (e.g., measles), which is why the calculations included the entire available time series. However, for the sake of completeness, Table S2 reports results for a shorter time period starting in December 2019. The results only differ marginally and do not change the classification of the correlations used in the study: no association (0.0 to 0.20), weak (0.20 to 0.40), moderate (0.40 to 0.60), and strong (>0.60).

	cross-dataset (all data)			intra-platform (all data, subset)	
Topic	Telegram, X	Telegram, news	X, news	Telegram	X
Death	0.36	0.29	0.36	0.49	0.71
Long COVID	0.29	0.13	0.03	0.33	0.59
Mandatory vaccination	0.88	0.65	0.66	0.76	0.85
Measures in schools	0.43	0.34	0.56	0.53	0.72
Virus variants	0.70	0.40	0.62	0.80	0.76

Table S2: Pearson correlation coefficients between Telegram, X, and mainstream news headlines datasets, considering time series between December 2019 and February 2023. Note: The same calculation procedure as for results in Table S1 was applied. The only difference is the shorter time series (starting in December 2019 instead of April 2019). Underlined cell values highlight different results compared to those reported in Table S1.

References

[1] World Health Organization. Coronavirus disease (COVID-19) pandemic. https://www.who.int/europe/emergencies/situations/covid-19, 2025. accessed 2025 Feb 24.
