# Supplementary material for: Information Pathways and Voids in Critical German Online Communities During the COVID-19 Vaccination Discourse: Cross-Platform and Mixed Methods Analysis
Source: J Med Internet Res. 2025 Oct 17;27:e76309. doi: 10.2196/76309 (PMC12557652; doi:10.2196/76309)
Supplement: Multimedia Appendix 4 [file jmir-v27-e76309-s004.pdf]

Multimedia appendix: Query

```sql
EXPORT DATA OPTIONS(
    uri=path_to_storage_location, -- e.g., gs://bucket/path/file_*.csv
    overwrite=TRUE,
    header=TRUE) AS
SELECT date, url, title
FROM gdelt-bq.gdeltv2.gemg
WHERE lang = 'GERMAN' AND date >= '2019-04-01' AND date < '2023-03-01'
```

Listing S1: Query used to retrieve GDELT data from Google BigQuery used in the analyses.
